# The genome sequence of the Broken-barred Carpet, *Electrophaes corylata *(Thunberg, 1792)

**DOI:** 10.12688/wellcomeopenres.19627.1

**Published:** 2023-06-28

**Authors:** Douglas Boyes, Owen T. Lewis, University of Oxford and Wytham Woods Genome Acquisition Lab, Darwin Tree of Life Barcoding collective, Wellcome Sanger Institute Tree of Life programme, Wellcome Sanger Institute Scientific Operations: DNA Pipelines collective, Tree of Life Core Informatics collective, Darwin Tree of Life Consortium

**Affiliations:** 1 UK Centre for Ecology & Hydrology, Wallingford, England, UK; 2 University of Oxford, Oxford, England, UK

**Keywords:** Electrophaes corylata, Broken-barred Carpet, genome sequence, chromosomal, Lepidoptera

## Abstract

We present a genome assembly from an individual male *Electrophaes corylata *(the Broken-barred Carpet; Arthropoda; Insecta; Lepidoptera; Geometridae). The genome sequence is 347.5 megabases in span. Most of the assembly is scaffolded into 30 chromosomal pseudomolecules, including the Z sex chromosome. The mitochondrial genome has also been assembled and is 16.36 kilobases in length. Gene annotation of this assembly on Ensembl identified 16,031 protein coding genes.

## Species taxonomy

Eukaryota; Metazoa; Eumetazoa; Bilateria; Protostomia; Ecdysozoa; Panarthropoda; Arthropoda; Mandibulata; Pancrustacea; Hexapoda; Insecta; Dicondylia; Pterygota; Neoptera; Endopterygota; Amphiesmenoptera; Lepidoptera; Glossata; Neolepidoptera; Heteroneura; Ditrysia; Obtectomera; Geometroidea; Geometridae; Larentiinae *; Electrophaes*; *Electrophaes corylata* (Thunberg, 1792) (NCBI:txid934936).

## Background

The Broken-barred Carpet ( *Electrophaes corylata*) is a small geometrid moth whose larvae feed on a variety of broad-leafed tree species. It occurs in a range of habitats including woodlands, gardens and hedgerows ( Henwood *et al.*, 2020; Waring *et al.*, 2017).

Although *E. corylata* is still very widespread and common throughout much of Britain and Ireland, there has been a significant decline in both its abundance and distribution since 1970 ( Randle *et al.*, 2019). Its global distribution extends across Europe and Asia to Japan ( GBIF Secretariat, 2023).

Here we present a chromosomally complete genome sequence for *E. corylata* based on one male specimen from Wytham Woods, Oxfordshire, UK. The genome of *E. corylata* was sequenced as part of the Darwin Tree of Life Project, a collaborative effort to sequence all named eukaryotic species in the Atlantic Archipelago of Britain and Ireland. A genome sequence for *E. corylata* will contribute to a growing data set of resources for understanding lepidopteran biology.

## Genome sequence report

**Figure 1.**
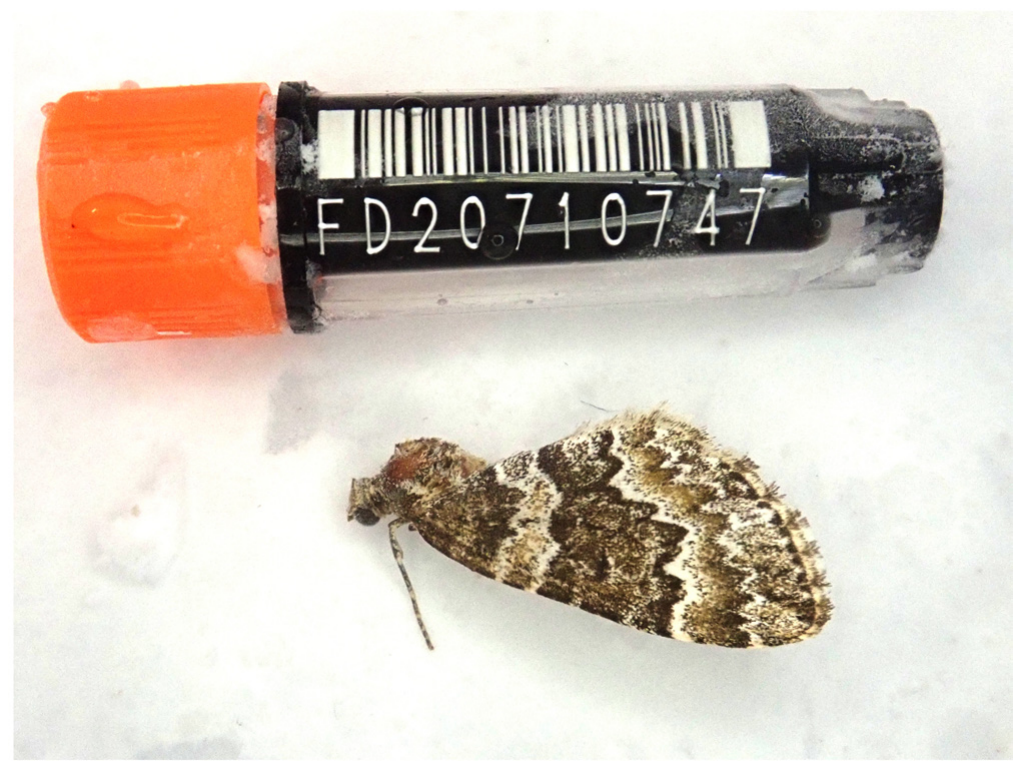
Photograph of the *Electrophaes corylata* (ilEleCory1) specimen used for genome sequencing.

The genome was sequenced from one male *Electrophaes corylata* ( Figure 1) collected from Wytham Woods, Oxfordshire, UK (51.77, –1.32). A total of 70-fold coverage in Pacific Biosciences single-molecule HiFi long reads was generated. Primary assembly contigs were scaffolded with chromosome conformation Hi-C data. Manual assembly curation corrected 3 missing joins or mis-joins and removed 3 haplotypic duplications, reducing the scaffold number by 7.89%.

**Figure 2.**
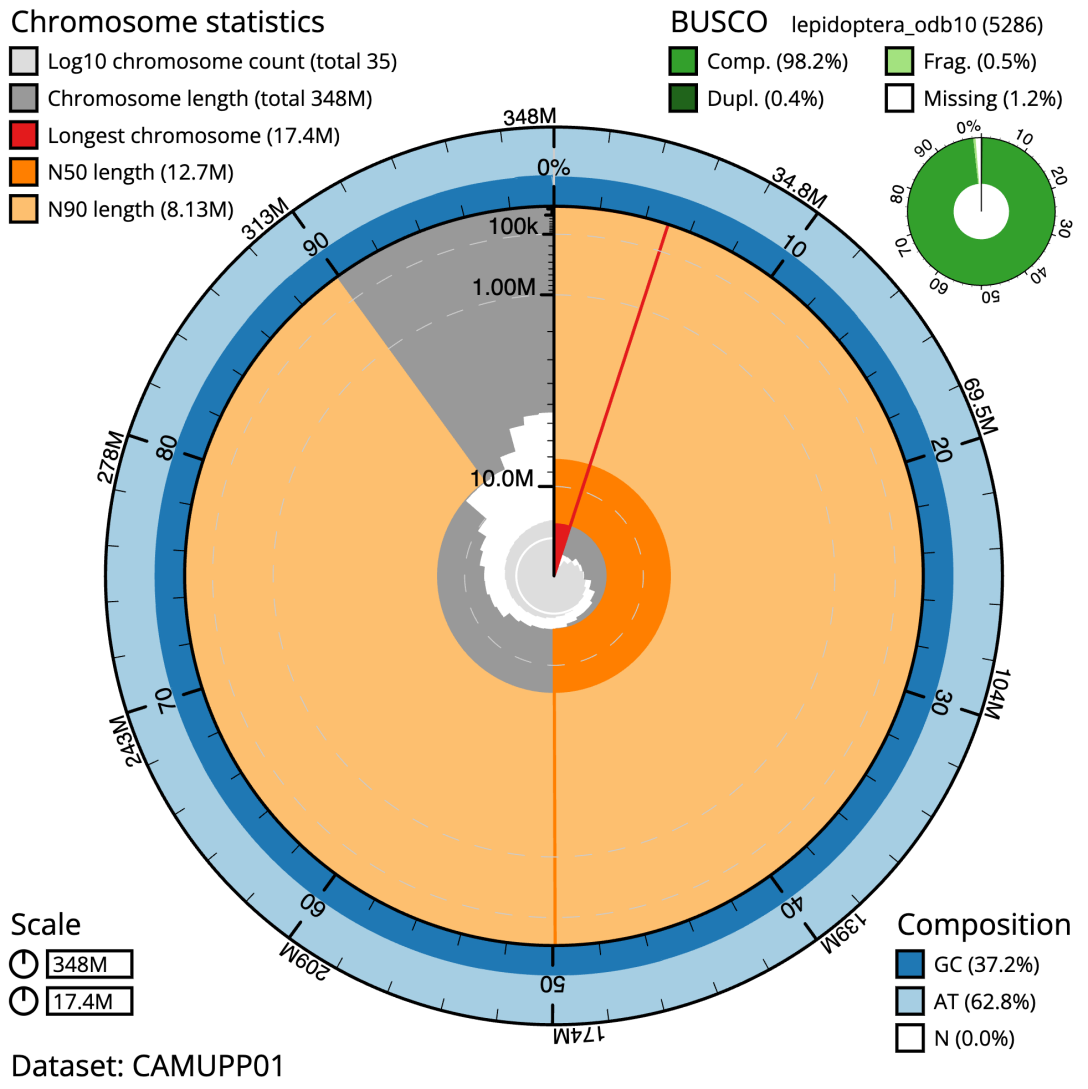
Genome assembly of *Electrophaes corylata*, ilEleCory1.1: metrics. The BlobToolKit Snailplot shows N50 metrics and BUSCO gene completeness. The main plot is divided into 1,000 size-ordered bins around the circumference with each bin representing 0.1% of the 347,554,160 bp assembly. The distribution of scaffold lengths is shown in dark grey with the plot radius scaled to the longest scaffold present in the assembly (17,402,341 bp, shown in red). Orange and pale-orange arcs show the N50 and N90 scaffold lengths (12,663,981 and 8,126,972 bp), respectively. The pale grey spiral shows the cumulative scaffold count on a log scale with white scale lines showing successive orders of magnitude. The blue and pale-blue area around the outside of the plot shows the distribution of GC, AT and N percentages in the same bins as the inner plot. A summary of complete, fragmented, duplicated and missing BUSCO genes in the lepidoptera_odb10 set is shown in the top right. An interactive version of this figure is available at https://blobtoolkit.genomehubs.org/view/Electrophaes corylata/dataset/CAMUPP01/snail

The final assembly has a total length of 347.5 Mb in 34 sequence scaffolds with a scaffold N50 of 12.7 Mb ( Table 1). Most (99.97%) of the assembly sequence was assigned to 30 chromosomal-level scaffolds, representing 29 autosomes and the Z sex chromosome. Chromosome-scale scaffolds confirmed by the Hi-C data are named in order of size ( Figure 2– Figure5; Table 2). While not fully phased, the assembly deposited is of one haplotype. Contigs corresponding to the second haplotype have also been deposited. The mitochondrial genome was also assembled and can be found as a contig within the multifasta file of the genome submission.

**Table 1.** Genome data for *Electrophaes corylata*, ilEleCory1.1.

Project accession data		
Assembly identifier	ilEleCory1.1	
Species	*Electrophaes corylata*	
Specimen	ilEleCory1	
NCBI taxonomy ID	934936	
BioProject	PRJEB56050	
BioSample ID	SAMEA10979139	
Isolate information	ilEleCory1, male: head and thorax (DNA sequencing and Hi-C scaffolding)	
Assembly metrics *		*Benchmark*
Consensus quality (QV)	66.6	*≥ 50*
*k*-mer completeness	100%	*≥ 95%*
BUSCO **	C:98.2%[S:97.9%,D:0.4%], F:0.5%,M:1.2%,n:5,286	*C ≥ 95%*
Percentage of assembly mapped to chromosomes	99.97%	*≥ 95%*
Sex chromosomes	Z chromosome	*localised homologous pairs*
Organelles	Mitochondrial genome assembled	*complete single alleles*
Raw data accessions		
PacificBiosciences SEQUEL II	ERR10224923	
Hi-C Illumina	ERR10297814	
Genome assembly		
Assembly accession	GCA_947095575.1	
*Accession of alternate haplotype*	GCA_947095555.1	
Span (Mb)	347.6	
Number of contigs	62	
Contig N50 length (Mb)	9.8	
Number of scaffolds	35	
Scaffold N50 length (Mb)	12.7	
Longest scaffold (Mb)	17.4	
Genome annotation		
Number of protein-coding genes	16,031	
Number of gene transcripts	16,210	

* Assembly metric benchmarks are adapted from column VGP-2020 of “Table 1: Proposed standards and metrics for defining genome assembly quality” from ( Rhie *et al.*, 2021). ** BUSCO scores based on the lepidoptera_obd10 BUSCO set using v5.3.2. C = complete [S = single copy, D = duplicated], F = fragmented, M = missing, n = number of orthologues in comparison. A full set of BUSCO scores is available at https://blobtoolkit.genomehubs.org/view/Electrophaes corylata/dataset/CAMUPP01/busco

**Figure 3.**
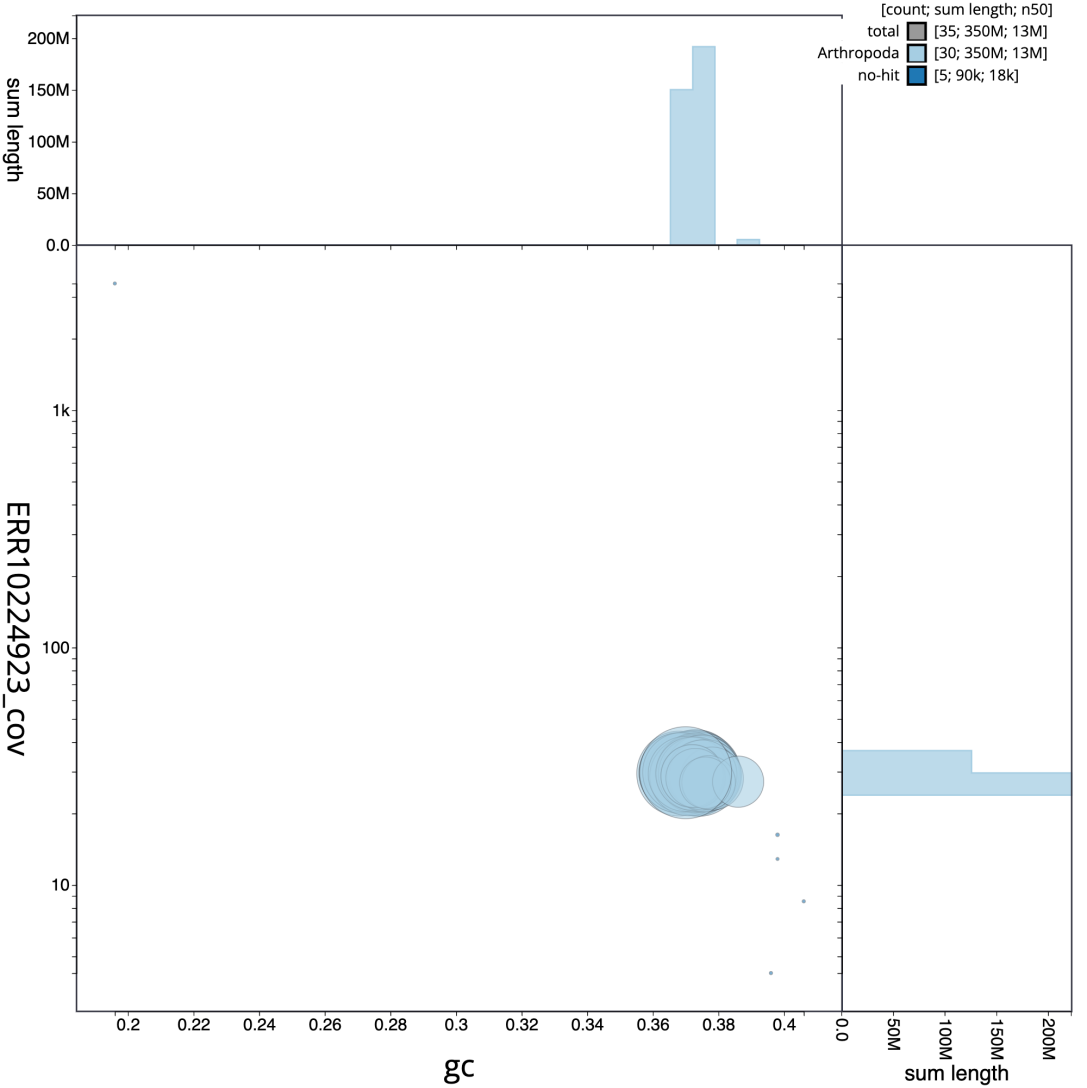
Genome assembly of *Electrophaes corylata*, ilEleCory1.1: BlobToolKit GC-coverage plot. Scaffolds are coloured by phylum. Circles are sized in proportion to scaffold length. Histograms show the distribution of scaffold length sum along each axis. An interactive version of this figure is available at https://blobtoolkit.genomehubs.org/view/Electrophaes corylata/dataset/CAMUPP01/blob.

**Figure 4.**
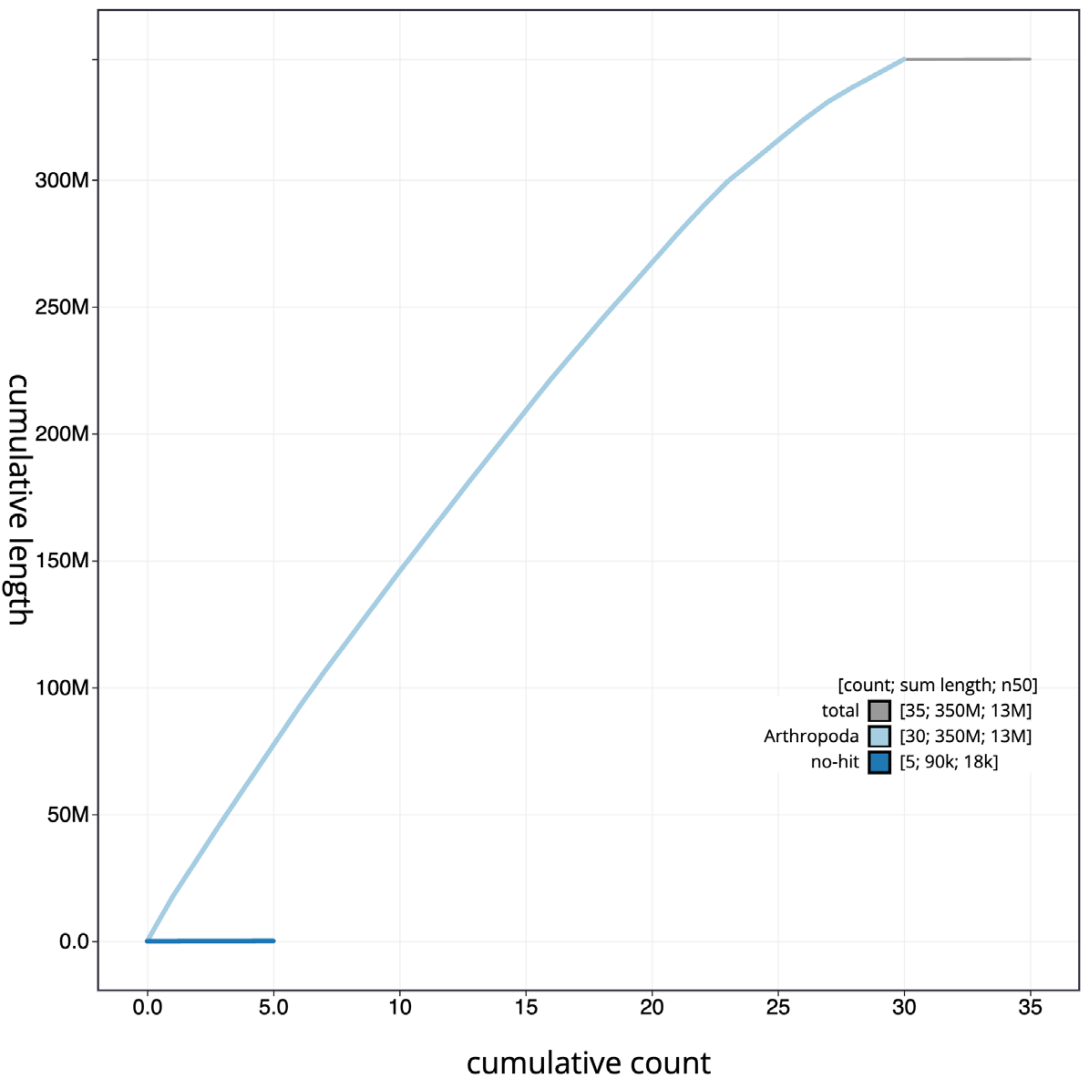
Genome assembly of *Electrophaes corylata* , ilEleCory1.1: BlobToolKit cumulative sequence plot.. The grey line shows cumulative length for all scaffolds. Coloured lines show cumulative lengths of scaffolds assigned to each phylum using the buscogenes taxrule. An interactive version of this figure is available at https://blobtoolkit.genomehubs.org/view/Electrophaes corylata/dataset/CAMUPP01/cumulative.

**Figure 5.**
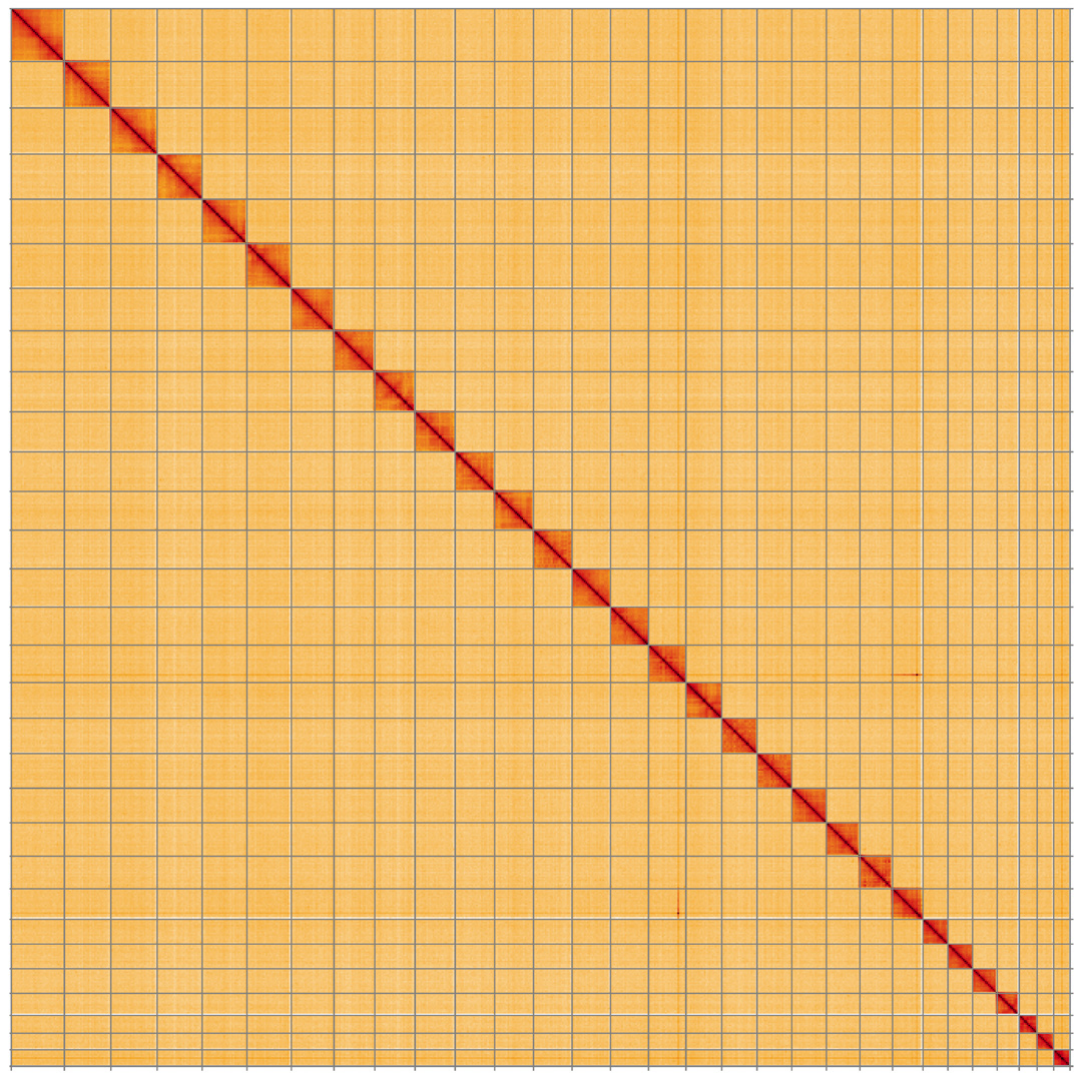
Genome assembly of *Electrophaes corylata*, ilEleCory1.1: Hi-C contact map of the ilEleCory1.1 assembly, visualised using HiGlass. Chromosomes are shown in order of size from left to right and top to bottom. An interactive version of this figure may be viewed at https://genome-note-higlass.tol.sanger.ac.uk/l/?d=SgopR3ypQ8mwld1Kv6Uerg.

**Table 2.** Chromosomal pseudomolecules in the genome assembly of *Electrophaes corylata*, ilEleCory1.

INSDC accession	Chromosome	Length (Mb)	GC%
OX352699.1	1	15.24	37.5
OX352700.1	2	15.19	37.5
OX352701.1	3	14.78	37.0
OX352702.1	4	14.69	37.5
OX352703.1	5	14.59	37.5
OX352704.1	6	13.96	37.0
OX352705.1	7	13.43	37.0
OX352706.1	8	13.19	37.0
OX352707.1	9	13.17	37.0
OX352708.1	10	12.98	37.0
OX352709.1	11	12.75	37.5
OX352710.1	12	12.66	37.0
OX352711.1	13	12.62	37.5
OX352712.1	14	12.47	37.5
OX352713.1	15	12.3	37.5
OX352714.1	16	11.73	37.5
OX352715.1	17	11.58	37.0
OX352716.1	18	11.41	37.5
OX352717.1	19	11.37	37.5
OX352718.1	20	10.97	37.0
OX352719.1	21	10.73	37.5
OX352720.1	22	10.01	37.5
OX352721.1	23	8.14	38.0
OX352722.1	24	8.13	37.0
OX352723.1	25	8.04	37.0
OX352724.1	26	7.24	37.5
OX352725.1	27	5.89	37.5
OX352726.1	28	5.44	37.5
OX352727.1	29	5.36	38.5
OX352698.1	Z	17.4	37.0
OX352728.1	MT	0.02	19.5

The estimated Quality Value (QV) of the final assembly is 66.6 with *k*-mer completeness of 100%, and the assembly has a BUSCO v5.3.2 completeness of 98.2% (single = 97.9%, duplicated = 0.4%), using the lepidoptera_odb10 reference set ( *n* = 5,286).

Metadata for specimens, spectral estimates, sequencing runs, contaminants and pre-curation assembly statistics can be found at https://links.tol.sanger.ac.uk/species/934936.

## Genome annotation report

The *Electrophaes corylata* genome assembly (GCA_947095575.1) was annotated using the Ensembl rapid annotation pipeline ( Table 1; https://rapid.ensembl.org/Electrophaes_corylata_GCA_947095575.1/Info/Index). The resulting annotation includes 16,210 transcribed mRNAs from 16,031 protein-coding genes.

## Methods

### Sample acquisition and nucleic acid extraction

The specimen used for genome assembly was a male *Electrophaes corylata* (specimen IDO x001879, individual ilEleCory1) was collected from Wytham Woods, Oxfordshire (biological vice-county Berkshire), UK (latitude 51.77, longitude –1.32) on 2021-05-28 using a light trap. The specimen was collected and identified by Douglas Boyes (University of Oxford) and preserved on dry ice.

The sample was prepared for DNA extraction at the Tree of Life laboratory, Wellcome Sanger Institute (WSI). The ilEleCory1 sample was weighed and dissected on dry ice with tissue set aside for Hi-C sequencing. DNA was extracted at the WSI Scientific Operations core using the Qiagen MagAttract HMW DNA kit, according to the manufacturer’s instructions.

### Sequencing

Pacific Biosciences HiFi circular consensus and DNA sequencing libraries were constructed according to the manufacturers’ instructions. DNA sequencing was performed by the Scientific Operations core at the WSI on a Pacific Biosciences SEQUEL II (HiFi) instrument. Hi-C data were also generated from head and thorax tissue of ilEleCory1 using the Arima2 kit and sequenced on the Illumina NovaSeq 6000 instrument.

### Genome assembly, curation and evaluation

Assembly was carried out with Hifiasm ( Cheng *et al.*, 2021) and haplotypic duplication was identified and removed with purge_dups ( Guan *et al.*, 2020). The assembly was then scaffolded with Hi-C data ( Rao *et al.*, 2014) using YaHS ( Zhou *et al.*, 2023). The assembly was checked for contamination and corrected as described previously ( Howe *et al.*, 2021). Manual curation was performed using HiGlass ( Kerpedjiev *et al.*, 2018) and Pretext ( Harry, 2022). The mitochondrial genome was assembled using MitoHiFi ( Uliano-Silva *et al.*, 2022), which runs MitoFinder ( Allio *et al.*, 2020) or MITOS ( Bernt *et al.*, 2013) and uses these annotations to select the final mitochondrial contig and to ensure the general quality of the sequence.

A Hi-C map for the final assembly was produced using bwa-mem2 ( Vasimuddin *et al.*, 2019) in the Cooler file format ( Abdennur & Mirny, 2020). To assess the assembly metrics, the *k*-mer completeness and QV consensus quality values were calculated in Merqury ( Rhie *et al.*, 2020). This work was done using Nextflow ( Di Tommaso *et al.*, 2017) DSL2 pipelines “sanger-tol/readmapping” ( Surana *et al.*, 2023a) and “sanger-tol/genomenote” ( Surana *et al.*, 2023b). The genome was analysed within the BlobToolKit environment ( Challis *et al.*, 2020) and BUSCO scores ( Manni *et al.*, 2021; Simão *et al.*, 2015) were calculated.

Table 3 contains a list of relevant software tool versions and sources.

**Table 3.** Software tools: versions and sources.

Software tool	Version	Source
BlobToolKit	4.0.7	https://github.com/blobtoolkit/ blobtoolkit
BUSCO	5.3.2	https://gitlab.com/ezlab/busco
Hifiasm	0.16.1-r375	https://github.com/chhylp123/ hifiasm
HiGlass	1.11.6	https://github.com/higlass/higlass
Merqury	MerquryFK	https://github.com/ thegenemyers/MERQURY.FK
MitoHiFi	2	https://github.com/marcelauliano/ MitoHiFi
PretextView	0.2	https://github.com/wtsi-hpag/ PretextView
purge_dups	1.2.3	https://github.com/dfguan/purge_ dups
sanger-tol/ genomenote	v1.0	https://github.com/sanger-tol/ genomenote
sanger-tol/ readmapping	1.1.0	https://github.com/sanger-tol/ readmapping/tree/1.1.0
YaHS	yahs- 1.1.91eebc2	https://github.com/c-zhou/yahs

### Genome annotation

The BRAKER2 pipeline ( Brůna *et al.*, 2021) was used in the default protein mode to generate annotation for the *Electrophaes corylata* assembly (GCA_947095575.1) in Ensembl Rapid Release.

### Wellcome Sanger Institute – Legal and Governance

The materials that have contributed to this genome note have been supplied by a Darwin Tree of Life Partner. The submission of materials by a Darwin Tree of Life Partner is subject to the **‘Darwin Tree of Life Project Sampling Code of Practice’**, which can be found in full on the Darwin Tree of Life website here. By agreeing with and signing up to the Sampling Code of Practice, the Darwin Tree of Life Partner agrees they will meet the legal and ethical requirements and standards set out within this document in respect of all samples acquired for, and supplied to, the Darwin Tree of Life Project.

Further, the Wellcome Sanger Institute employs a process whereby due diligence is carried out proportionate to the nature of the materials themselves, and the circumstances under which they have been/are to be collected and provided for use. The purpose of this is to address and mitigate any potential legal and/or ethical implications of receipt and use of the materials as part of the research project, and to ensure that in doing so we align with best practice wherever possible. The overarching areas of consideration are:

•   Ethical review of provenance and sourcing of the material

•   Legality of collection, transfer and use (national and international) 

Each transfer of samples is further undertaken according to a Research Collaboration Agreement or Material Transfer Agreement entered into by the Darwin Tree of Life Partner, Genome Research Limited (operating as the Wellcome Sanger Institute), and in some circumstances other Darwin Tree of Life collaborators.

## Data Availability

European Nucleotide Archive: *Electrophaes corylata* (broken-barred carpet). Accession number PRJEB56050; https://identifiers.org/ena.embl/PRJEB56050. ( Wellcome Sanger Institute, 2022) The genome sequence is released openly for reuse. The *Electrophaes corylata* genome sequencing initiative is part of the Darwin Tree of Life (DToL) project. All raw sequence data and the assembly have been deposited in INSDC databases. Raw data and assembly accession identifiers are reported in Table 1.

## Author information

Members of the University of Oxford and Wytham Woods Genome Acquisition Lab are listed here: https://doi.org/10.5281/zenodo.4789928.

Members of the Darwin Tree of Life Barcoding collective are listed here: https://doi.org/10.5281/zenodo.4893703.

Members of the Wellcome Sanger Institute Tree of Life programme are listed here: https://doi.org/10.5281/zenodo.4783585.

Members of Wellcome Sanger Institute Scientific Operations: DNA Pipelines collective are listed here: https://doi.org/10.5281/zenodo.4790455.

Members of the Tree of Life Core Informatics collective are listed here: https://doi.org/10.5281/zenodo.5013541.

Members of the Darwin Tree of Life Consortium are listed here: https://doi.org/10.5281/zenodo.4783558.
