# Supplementary material for: The genome sequence of the Broken-barred Carpet, Electrophaes corylata (Thunberg, 1792)
Source: Wellcome Open Res. 2023 Jun 28;8:283. [Version 1] doi: 10.12688/wellcomeopenres.19627.1 (PMC10521117; doi:10.12688/wellcomeopenres.19627.1)
Supplement: Peer review history

# Reviewer report: Reviewer response for version 1

**DOI:** 10.21956/wellcomeopenres.21743.r65862

**Published:** 2023-09-18

**Authors:** Xue-Yan Li

**Affiliations:** 1 Chinese Academy of Sciences, Kunming, China

This MS provides a high-quality genome assembly of geometrid moth *Electrophaes corylata *from an individual male. This is very important resources for lepidopteran genomes. Nevetheless, I hope the authors to give more backbround about the species, especially if it take some damages agriculture, and forestry, considering its wide range of habitats including for woodlands, gardens and hedgerows.

Are sufficient details of methods and materials provided to allow replication by others?

Yes

Is the rationale for creating the dataset(s) clearly described?

Yes

Are the datasets clearly presented in a useable and accessible format?

Yes

Are the protocols appropriate and is the work technically sound?

Yes

Reviewer Expertise:

Insect genome

I confirm that I have read this submission and believe that I have an appropriate level of expertise to confirm that it is of an acceptable scientific standard.

# Reviewer report: Reviewer response for version 1

**DOI:** 10.21956/wellcomeopenres.21743.r65866

**Published:** 2023-09-11

**Authors:** Simon H Martin

**Affiliations:** 1 University of Edinburgh, Edinburgh, UK

This report describes a chromosome-scale genome assembly for the Broken-barred Carpet, *Electrophaes corylata*, as part of the Darwin Tree of Life initiative.

 The assembly was produced using world leading approaches: PacBio Hi-Fi reads and scaffolding with Hi-C. The resulting assembly is of excellent quality by all metrics. An annotation was performed based on homology alone (RNA-seq data was no used), but it appears to have very high accuracy.

 I have no concerns about the manuscript or the resource described.

Are sufficient details of methods and materials provided to allow replication by others?

Yes

Is the rationale for creating the dataset(s) clearly described?

Yes

Are the datasets clearly presented in a useable and accessible format?

Yes

Are the protocols appropriate and is the work technically sound?

Yes

Reviewer Expertise:

Evolutionary genomics, population genomics

I confirm that I have read this submission and believe that I have an appropriate level of expertise to confirm that it is of an acceptable scientific standard.
